# Cold Atmospheric Plasma: Pre- and Post-Packaging Application for Fresh-Cut Apple Preservation

**DOI:** 10.3390/foods15132288

**Published:** 2026-06-26

**Authors:** Gabriela Inés Denoya, María Eugenia Novillo, Nancy Mariel Apóstolo, Gustavo Alberto Polenta, Mariano Manuel Fernández, Diego Sebastián Cristos, Ezequiel Cejas, Brenda Lorena Fina, Leandro Prevosto, Sergio Ramón Vaudagna

**Affiliations:** 1 Instituto Nacional de Tecnología Agropecuaria (INTA), Instituto Tecnología de Alimentos, Nicolás Repetto y De Los Reseros s/n, Hurlingham 1686, Buenos Aires, Argentina; novillo.maria@inta.gob.ar (M.E.N.); polenta.gustavo@inta.gob.ar (G.A.P.); cristos.diego@inta.gob.ar (D.S.C.); vaudagna.sergio@inta.gob.ar (S.R.V.); 2 Instituto de Ciencia y Tecnología de Sistemas Alimentarios Sustentables, UEDD INTA CONICET, Nicolás Repetto y De Los Reseros s/n, Hurlingham 1686, Buenos Aires, Argentina; 3 Consejo Nacional de Investigaciones Científicas y Técnicas (CONICET), Av. Rivadavia 1917, Ciudad Autónoma de Buenos Aires C1033AAJ, Argentina; cejasezequiel87@gmail.com (E.C.); brendafina@gmail.com (B.L.F.); leandroprevosto@gmail.com (L.P.); 4 Departamento de Ciencias Básicas, Universidad Nacional de Luján, CC 221, Luján 6700, Buenos Aires, Argentina; nancy.apostolo2@gmail.com; 5 Instituto Nacional de Tecnología Agropecuaria (INTA), EEA Pergamino, AERSan Antonio de Areco, Zapiola 237, San Antonio de Areco 2760, Buenos Aires, Argentina; fernandez.mariano@inta.gob.ar; 6 Grupo de Descargas Eléctricas, Departamento Ing. Electromecánica, Facultad Regional Venado Tuerto, Universidad Tecnológica Nacional (UTN), Laprida 651, Venado Tuerto 2600, Santa Fe, Argentina

**Keywords:** cold plasma, non-thermal, quality, apple, sugars, antioxidants, microstructure

## Abstract

Preserving minimally processed fruits represents a technological challenge. Therefore, non-thermal plasma (NTP) is proposed as one of the preservation methods. The aim of this work was to evaluate the effect of the application of NTP before and after packaging sliced apples in two films: One with high and another with low oxygen barrier features. Different parameters were evaluated during 14 days at 4 °C. Samples treated before packaging showed lower crunchiness and browning development, as indicated by chromatic and textural parameters. NTP reduced mesophiles, psychrotrophs, and yeasts and molds on the slices by 1–2 log units, although it had no effect on the antioxidant content of the apple slices, which were better preserved with high-barrier packaging. Samples treated with NTP, and low barrier packaging showed lower sugar content on day one. When applied after packaging, NTP contributed to better preservation of slice microstructure and tissue viability. Results showed that the combination of NTP applied after packaging and high-barrier film was the most suitable for maintaining fruit quality, mainly by better preserving slice color. In turn, tissue microstructure, texture analysis, and viability tests also supported this conclusion.

## 1. Introduction

Apple (*Malus domestica*) is one of the most economically relevant fruits produced in the world, whose industrialization rate has increased during the last decades [1]. On the other hand, consumers demand convenient products like fresh-cut fruit, because these products are either ready-to-eat or easy to incorporate into culinary preparations, also representing an indispensable input for restaurants and catering companies [2]. The application of novel technologies capable of preserving the safety and quality of fresh-cut fruit will be of utmost importance for the development of alternatives with extended shelf life [3]. Among them, non-thermal plasma (NTP) technology represents a promising alternative because of its effectiveness to disinfect food surfaces due to its low penetrability, this treatment generally does not affect their quality.

NTP technology is based on physicochemical phenomena. Matter generally exists in three states—solid, liquid, and gas—transitioning between them upon the addition of energy. When a gas is further energized to the point of ionization, a fourth state of matter, known as plasma, is generated. Thus, plasma can be generated by ionizing a gas at different pressures (high, low, or atmospheric). In air or similar gas mixtures, this process generates highly reactive oxygen and nitrogen species (ROS and RNS), such as O_3_, OH, NO, O_2_^−^, and NO_2_, as well as UV photons and other charged particles, which exhibit significant antimicrobial activity [4].

When a gas is highly ionized, its electrons and heavy species have very similar temperatures, and the plasma approaches thermal equilibrium. However, when the degree of ionization is low, the electrons absorb most of the energy from the electric field because they cannot efficiently transfer it to the gas. As a result, non-thermal plasma (NTP) is generated, with highly energetic electrons while the gas remains close to room temperature [5]. Several plasma generation methods have been described, among them micro-plasma jets, corona, glow, radiofrequency microwave, and dielectric barrier discharge (DBD). The latest is suitable for in-package processing, allowing the direct generation of reactive oxygen/nitrogen species within the sealed packages. This approach avoids the risk of post-process contamination. The associated microbial inactivation efficacy can be influenced by the packaging parameters, including the packaging material and the composition of the headspace inside the package. Interestingly, NTP could be used either to improve specific quality parameters or to develop novel products [6].

The inherent physical and chemical properties of the substrate are the main determinants of the outcome. In particular, attributes such as product geometry and morphology strongly influence the plasma–surface interface. Therefore, a thorough understanding of these intrinsic factors in each case is essential to optimize treatment efficiency and ensure uniform application [7].

Operating under thermodynamic non-equilibrium, atmospheric plasma technology provides a high-efficiency, low-energy solution for surface treatment. Its ability to selectively inactivate surface microorganisms and enzymes—without penetrating the sample matrix—prevents the degradation of nutritional and sensorial properties, potentially leading to high quality products. Furthermore, the absence of derived chemical residues highlights its potential as a safe and sustainable intervention [8]. In this sense, previous studies analyzed changes in the morphology and surface chemical composition of cold atmospheric plasma-treated seeds by using a battery of diagnostic techniques (X-ray Photoelectron Spectroscopy, Scanning Electron Microscopy—Energy Dispersive X-ray Spectroscopy, and Attenuated Total Reflection—Fourier Transform Infrared Spectroscopy); the results showed no toxic residues on the seeds [9]. Moreover, since the reactive species generated by NTP have lifetimes ranging from nanoseconds to hours, no residues of these species remain on treated products [10].

Although the use of NTP technology for preserving fresh-cut fruit has been assessed in previous studies [6,11], research on the effect of this technology on the physicochemical and nutritional parameters of the products due to the interaction of reactive species with the components is still limited [12]. Moreover, a significant research gap still exists regarding the combined influence of different packaging materials and the timing of NTP application on the various quality characteristics of these products. In addition, the effects of NTP on important aspects such as tissue microstructure, tissue viability, and relevant quality indicators (e.g., sugar content and ascorbic acid) are not usually considered. Therefore, the objective of this study was to compare the effectiveness of NTP applied before or after packaging, in combination with two different packaging films, for maintaining quality and extending the shelf life of minimally processed apples.

## 2. Materials and Methods

### 2.1. Plant Material and Processing

Apples (*Malus domestica*) var. Granny Smith were acquired from a market in Buenos Aires, Argentina. The fruits were selected at their optimum ripening stage for commercial distribution, ensuring the integrity of their physicochemical properties. For this assay, approximately 120 fruits were selected for processing and stored at 0 °C until the day of the assay. Apples were washed with tap water, and the central cores were removed using a stainless-steel apple corer. The fruits were subsequently sectioned into 0.5 cm thick slices using a commercial electric food slicer (Ultracomb, Buenos Aires, Argentina). Immediately afterwards, the pieces were immersed for 1 min in a 0.5% ascorbic acid (aq.) solution to avoid enzymatic browning during processing. Samples were packaged in two different films: (H) High oxygen barrier (6–14 cm^3^/m^2^/24 hs-Cryovac-BB2620) and (L) low oxygen barrier (1536 cm^3^/m^2^/24 hs-Resinite). The high oxygen barrier packaging produces a modified atmosphere inside the packaging with a reduced O_2_ concentration (8–10%) and a higher CO_2_ concentration (14–16%) that was proven effective for preserving fresh-cut peaches in our previous work [13]. The low oxygen barrier packaging maintains the composition similar to the ambient air (20% O_2_ and 0.5% CO_2_). Each package with nine apple slices was defined as the experimental unit. In turn, control samples were similarly prepared (CH and CL, for film of high and low barrier to oxygen respectively). Then, the NTP treatment was applied for 1 min on samples, either before (BH and BL) or after packaging (AH and AL), and with a high or low barrier to oxygen films respectively. The time of exposure to the NTP treatment was based on our previous work [14]. After the treatments, the samples were stored at 4 °C for 14 days. The treatments are summarized in Table 1.

### 2.2. DBD Plasma Source

The DBD system used in this work was the same as previously described in detail [14]. Briefly, it consisted of a dielectric barrier–stabilized corona discharge with a needle-array high-voltage electrode and a plate ground electrode covered by a dielectric barrier of three polyester films (400 μm thick Thernophase).

For the treatment application, six packages containing the apple samples were placed between the two electrodes. The atmosphere inside the packages was ambient air. In the case of the in-package (after packaging) treatments, the film of the package also acted as a dielectric barrier. The gap between the electrodes was fixed at 20 mm during the experiments. The corresponding distance between the needles and the samples (inside the packages) was about 14 mm. All treatments were conducted in ambient air (without superimposed gas flow) using a high-voltage sine AC power supply operating at 50 Hz. A voltage of 30 kV (peak value) was applied across the electrodes during the treatments. The discharge power was inferred by using the Lissajous plot method, employing a 33 μF capacitor connected in series with the grounded discharge electrode to measure the transported electric charge. The measured discharge power values ranged from 50 to 60 W. Additionally, ozone concentration levels within the package were quantified via UV-Vis absorption spectroscopy, following a configuration similar to that reported previously [15]. Ozone concentration was determined from the absorbance measured at a wavelength of 253.65 nm, applying the Lambert–Beer law. The absorption cross section used for calculations was 1.14 × 10^−21^ m^2^ for a gas temperature of 300 K. The peak ozone concentration at 1 min was close to 150 ppm. No presence of nitrogen oxides could be detected inside the packages. The rise in surface temperature of the dielectric barrier did not exceed 10 °C during the plasma exposure time. For each treatment, three replicates were prepared by placing the experimental units of each replicate within the active plasma region on the dielectric barrier.

### 2.3. Experimental Design and Statistical Analysis

A completely randomized design with a 3 × 2 × 3 factorial arrangement was used (*n* = 3). The independent variables were the application of the DBD Plasma treatment (control (C)—without NTP treatment—, application of NTP before (B) or after packaging (A)), the packaging film—low (L) and high (H) barrier to oxygen—and the storage times at 4 °C (1, 7 and 14 days). Three slices from each of three experimental units (3 containers) from each treatment and per day of storage were analyzed to carry out microbiological analysis, fracturability, and color determinations. The extracts to carry out biochemical determinations (total phenols content, antioxidant capacity, vitamin C and sugars) were prepared with two-three slices from each of these experimental units. Microstructure analysis was performed using micrographs obtained from different experimental units stored for 4 days at 4 °C. This allowed for the evaluation of tissue preservation in both treated and untreated samples.

The means and standard errors of the experimental values for each treatment were calculated. Differences were tested for significance by analysis of variance, which was performed using the General Linear Model procedure from SAS ONDEMAND FOR ACADEMICS 3.82 Enterprise Edition (SAS Institute Inc., University Edition, Cary, NC, USA). Duncan’s test (significance level of 0.05) was performed for the data presented in tables and graphics.

### 2.4. Sample Analysis

#### 2.4.1. Microbiological Analysis

All samples were serially diluted with a sterile 0.1% *w*/*v* peptone solution, and 1 mL of each dilution was pour-plated into the corresponding medium. A Plate Count Agar medium (PCA, Oxoid, UK) was used to determine the total aerobic mesophilic (RAM) counts after incubation at 37 °C for 48 h and psychotropic counts after incubation at 4 °C for 15 days. In the case of molds and yeast, the medium used was Yeast Extract Dextrose Chloramphenicol (YEDC, Oxoid, UK). The incubation was at 20 °C for 5 days. In all the cases, grown microbial colonies were enumerated, and the microbial load of each sample was expressed as log colony forming units (CFU) per gram of apples (log CFU g^−1^).

#### 2.4.2. Chromatic Parameters

Chromatic parameters of three fruit pieces (three points on each slice) from each experimental unit were measured with a Minolta CR-400 chromameter (Konica Minolta Sensing, Inc., Osaka, Japan). Results were expressed in CIE L*a*b* color space. The instrument was set up for illuminant D_65_ and 2° observer angle. Browning index (BI) for each sample was calculated from the values obtained for L*, a* and b* with the following formula according to [16]: BI = [[100 (x − 0.310)]/0.172] were x is equal to

x = (a* + 1.75 L*)/(5.645 L* + a* − 3.012 b*)

#### 2.4.3. Fracturability

The fracturability of the apple slices was performed by carrying out a puncture test with a TA-XT plus Texture Analyzer (Stable Micro Systems Ltd., Surrey, UK) with a cylindrical probe of 2 mm of diameter and a cell load of 5 kg. Three slices of each experimental unit were individually measured in three different points. The conditions were: Velocity of the probe during the test: 1 mm s^−1^ and the distance penetrated by the probe inside the sample was set in 3 mm. Fracturability (dimensionless) was measured as the length after linearization of the curve of force vs. time [17].

#### 2.4.4. Soluble Solids

Soluble solid content (SSC) was determined at 20 °C by measuring the refractive index of the juice of the apple slices with a refractometer (Atago Co., Ltd., Tokyo, Japan).

#### 2.4.5. Extraction for Total Phenol and Sugar Content and Antioxidant Capacity

The extraction for total phenol and sugar content and antioxidant capacity determinations was done according to [18].

- Total phenols content determination

The total phenol content determination was carried out according to [19], adapted for its realization in microplate wells [18]. The content of total phenols was expressed as grams of gallic acid equivalents (GAE) per kilogram. Results were expressed on a fresh weight basis.

- Antioxidant capacity

The antioxidant capacity was determined on the extracts based on three methods: ABTS and DPPH (electron and radical scavenging assay, respectively) and Ferric reducing/antioxidant power (FRAP), focused on reducing/oxidizing ability of the extracts. The antioxidant capacity was carried out according to [18]. The antioxidant capacity was expressed in Trolox (6-hydroxy-2,5,7,8-tetramethylchroman-2-carboxylic acid) equivalents (TEAC): mM eq. Trolox per kilogram. Results were expressed on a fresh weight basis.

- Sugar content

The sugar content of the samples, in particular the concentration of sucrose, D-glucose and D-fructose was determined using a commercial enzymatic kit (Sucrose/D-glucose/D-fructose Enzymatic BioAnalysis, Boehringer Mannheim, R-Biopharm, Darmstadt, Germany) according to the manufacturer recommendations. Results were expressed as g of sugar per kg of fresh fruit.

#### 2.4.6. Ascorbic Acid

- Extraction

The extraction of ascorbic acid was performed according to the technique described by [20] with some modifications. Samples were crushed with a Homogenizer HH-S-1000 (Hermann, Germany) at 30,000 rpm for 2 min on slush ice and protected from light. Two grams of each homogenate were weighed and mixed with 5 mL of an aqueous solution containing 10% (*w*/*v*) perchloric acid (HClO_4_) and 1% (*w*/*v*) metaphosphoric acid (HPO_3_). The mixture was vortexed for 2 min and then centrifuged for 10 min at 10,000× *g* at 4 °C. The supernatant was separated and then filtered through a 0.22 µm nylon filter and immediately analyzed.

- HPLC Determination

The content of ascorbic acid present in the samples was determined by using reversed-phase high performance liquid chromatography (HPLC) on an Alliance-2695 Separations Module chromatograph (Waters™, Milford, MA, USA) equipped with a quaternary solvent delivery system and a 2996 photodiode array (PDA). The column used was C_18_ Pheno Sphere-NEXT, 5 µm, 4.6 mm ID × 150 mm, 120 Å (Thermo Fisher Scientific Inc., Waltham, MA, USA). Solution “A” (0.01 M potassium phosphate buffer, pH 2.5) and solution “B” (methanol) were used as mobile phase. An isocratic flow was used during the run time: 85% solution “A” and 15% solution “B”. The flow rate was 0.5 mL/min. The injection volume was 10 µL per sample and the reading wavelength was 243 nm. Quantification was based on an external calibration curve, and results were expressed as milligrams of ascorbic acid per 100 g of fresh tissue (mg/100 g FW).

#### 2.4.7. Light Microscopy

Light microscopy was determined according to [14]. These observations were made on tissue samples taken from the surface and the inner area of the slices.

#### 2.4.8. Viability Test

Viability assay was determined according to [21]. These observations were made on tissue samples taken from the surface and the inner area of the slices.

## 3. Results and Discussion

### 3.1. Microbiological Analysis

**Figure 1:**
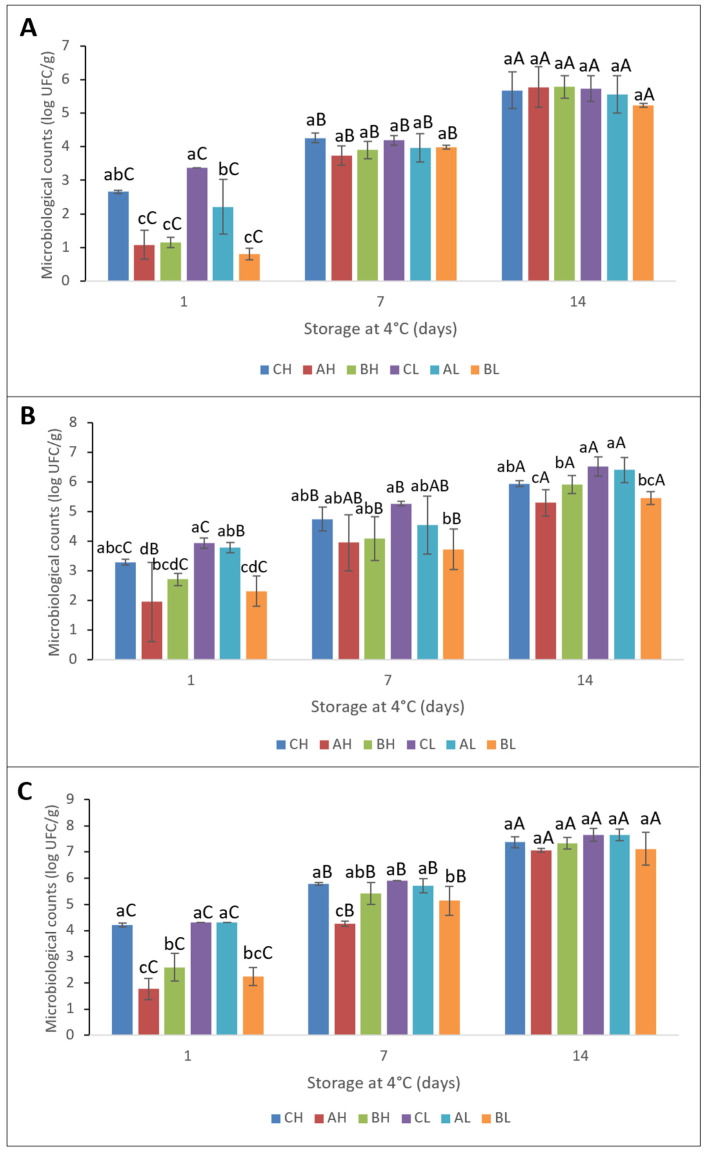
(**A**) Total aerobic mesophilic (RAM), (**B**) molds and yeast, and (**C**) psychotropic counts of minimally processed apples treated at different non-thermal plasma (NTP) treatments during storage at 4 °C. Data are expressed as means (*n* = 3) and error bars correspond to standard deviations. For each storage time, the means at different treatment followed by the same lowercase letter were not significantly different according to Duncan’s test (*p* = 0.05). For each plasma treatment, means at different storage time followed by the same uppercase letter were not significantly different according to Duncan’s test (*p* = 0.05).

Figure 1 shows the microbiological counts of minimally processed apples subjected to the different NTP treatments during 14 days of refrigerated storage. Total aerobic mesophilic viable counts (Figure 1A) were significantly reduced (*p* < 0.05) in all treated samples by 1–2 log units on day 1, regardless of the mode of NTP application. The reduction was lower only in slices treated after packaging with the low-barrier film (AL). On day seven of refrigerated storage, a non-significant trend toward lower counts was observed in treated samples compared with control samples. In all treatments, microbial counts increased steadily during refrigerated storage, and after 14 days all samples reached similar count levels.

In a previous study, a similar behavior was observed in peaches treated at 30 kV for 4 min, where the aerobic mesophilic counts were significantly reduced by 1.67 log at day zero, with counts tending to attain lower values than the control during the storage at 20 °C [22]. In strawberries, Ref. [23] found a one log reduction immediately after the application of an in-package treatment (60 kV for 10 min), although the difference with the untreated samples was not maintained during storage. In blueberries, DBD treatment at 4 kV for 20 min reduced aerobic mesophilic counts by 0.59 log units [24]. The authors explained that the main antimicrobial agents in NTP are reactive oxygen and nitrogen species, which can induce oxidative stress in microbial cells, leading to oxidative damage to membranes and intracellular components, and ultimately to cell death.

However, a different trend was observed by [25] in fresh-cut mangoes, where DBD treatment at 75 kV for 3 min did not produce an immediate reduction after application, but markedly restricted microbial growth by causing lethal injury. As a result, treated fruit showed reductions of 1.04 and 0.61 log units in total microbial counts after 2 and 4 days of storage, respectively. In another study, strawberry wedges subjected to in-package treatment (45 kV for 1 min at 20 °C) showed a significant reduction in aerobic mesophilic counts from day 3 at 4 °C, reaching a 1 log reduction on day 7 compared with untreated sample [3].

In the case of yeasts and molds (Figure 1B), a 1–2 log reduction was also observed in NTP-treated samples on day 1, although the greatest reductions were found for AH and BL. On day 7, a similar trend was observed, but BL was the only treatment that significantly reduced mold counts compared with the control samples. By day 14, treated samples generally showed lower counts than the controls, with AH and BL showing significantly lower counts (0.8 and 1.2 log reductions compared with the controls, respectively). Regardless of the treatment, counts increased throughout refrigerated storage.

In whole yellow peaches treated at 30 kV for 4 min, the reduction in yeasts and molds was not statistically significant on day 0 (0.98 log), although it became significant during 16 days of storage at 20 °C [22]. In blueberries, DBD treatment at 4 kV for 20 min reduced fungal counts by 0.7 log units immediately after treatment compared with the control, and these lower counts were maintained during storage at 25 °C, reaching a 1.8 log reduction after 10 days [24]. In addition, a 0.59 log reduction was reported by [26] for blueberries treated at 36 V and 1.8 A for 10 min.

For psychrotrophs (Figure 1C), significant 2-log unit reductions with respect to control samples were observed for the AH and BL treatments at days 1 and 7, and for the BH treatment only on day 1. In turn, on day 14, all treatments presented similar counts.

For all treatments, counts increased along with the days of refrigerated storage.

It is known that most bacteria are very sensitive to reactive oxygen species. Thus, bombardment with charged particles may lead to cell wall surface lesions, a process known as etching, which induces further penetration of plasma-toxic compounds inside the microbial cells. This eventually leads to the rupture of the cell membrane by a process called electropermeabilization. Microbial inactivation by NTP is currently considered to result from physicochemical, chemical, and biological interactions between reactive species and bacterial cell membranes. Reactions involving oxygen radicals can cause the oxidation of cellular DNA, lipid peroxidation, and protein denaturation through amino acid oxidation. In turn, reactive nitrogen species can accumulate on the microbial surface and readily diffuse through cell membranes, causing a decrease in intracellular pH. This reduction in intracellular pH may affect enzymes, other proteins, nucleic acids, and reaction rates. In addition to reactive species, UV photons may also interfere with cell replication by altering microbial DNA. However, UV photons in NTP appear to play only a minor role in microbial inactivation [11,27,28].

Although all treatments reached similar counts by the end of storage, in the case of psychrotrophs and yeasts and molds, the lowest counts, up to day 7 of refrigerated storage, were observed for NTP applied after packaging with the high oxygen-barrier film (AH) and before packaging with the low oxygen-barrier film (BL).

### 3.2. Chromatic Parameters

**Figure 2:**
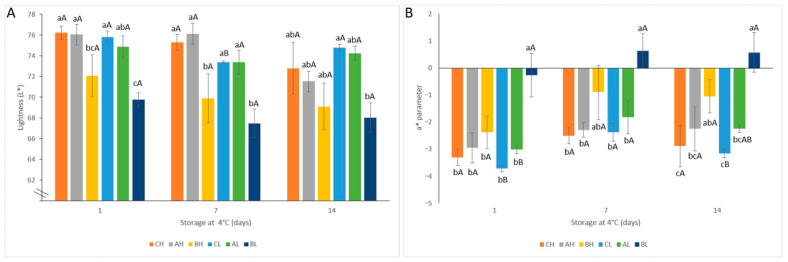
Color parameters: (**A**) L* and (**B**) a*, of minimally processed apples treated at different non-thermal plasma (NTP) treatments during storage at 4 °C. Data are expressed as means (*n* = 3) and error bars correspond to standard deviations. For each storage time, the means at different treatment followed by the same lowercase letter were not significantly different according to Duncan’s test (*p* = 0.05). For each plasma treatment, means at different storage time followed by the same uppercase letter were not significantly different according to Duncan’s test (*p* = 0.05).

The chromatic parameters of samples subjected to the different treatments are shown in Figure 2, and the browning indices are presented in Table S1. In apple products, a decrease in L* accompanied by an increase in a* is strongly associated with greater browning [29]. Samples treated with NTP before packaging (BL and BH) presented the lowest values of lightness (L*) and, in the case of the BL samples, the highest values of a* and browning index, along the entire refrigerated storage. This evidenced a major induction of enzymatic browning in these samples. In the case of browning index, significant differences between the treatments were observed on day seven, with AH, CH, and AL being the ones with the lower values. A previous study by [30] also reported changes in L* (decrease) and a* (increase) values of apples cubes after the direct application (without package) of a DBD-treatment at 20 kV 15 min, although these were considered imperceptible. These authors found major changes in color when applying the treatment to apple juice. Ref. [31] also informed lower L* and higher a* values for jackfruit slices with a DBD-treatment at 6 kV, 25 kHz, up to 1 min as a pre-treatment for the drying process in comparison to untreated samples.

No other significant differences in chromatic parameters were found for the rest of the treatments, regardless of the day of evaluation, which evidence that sample color was well preserved during the 14 days of refrigerated storage. Then, the samples treated after packaging and those having high barrier to oxygen packaging were the ones with less induction of enzymatic browning. In a previous study, we proved that apple slices treated with a DBD-treatment at 30 kV 50 Hz for 1 and 3 min after packaging adequately maintained L* and a* values during the refrigerated storage [14]. Moreover, we measured the polyphenol oxidase activity of the samples, one of the main enzymes responsible for catalyzing browning in fruits, and observed a 20% reduction after the 1 min treatment, which was the same treatment applied to the AH samples. On the other hand, Ref. [32] reported a browning effect of different in-package cold atmospheric plasma treatments (45–65 kV for 1–5 min) on fresh-cut pears, especially after 5 and 7 days of refrigerated storage. In our study, this effect was mitigated by an immersion step in a 0.5% ascorbic acid solution, which prevented the subsequent browning of the samples during storage.

### 3.3. Fracturability

**Figure 3:**
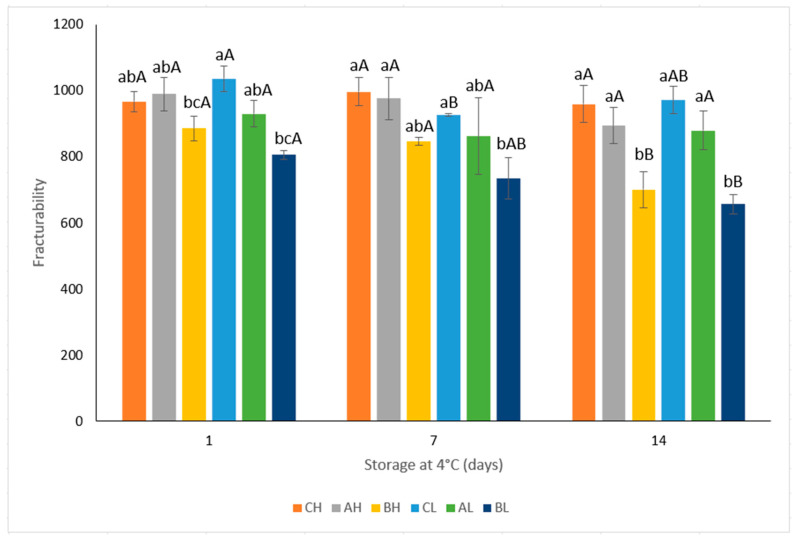
Fracturability (dimensionless) of minimally processed apples treated at different non-thermal plasma (NTP) treatments during storage at 4 °C. Data are expressed as means (*n* = 3) and error bars correspond to standard deviations. For each storage time, the means at different treatment followed by the same lowercase letter were not significantly different according to Duncan’s test (*p* = 0.05). For each plasma treatment, means at different storage time followed by the same uppercase letter were not significantly different according to Duncan’s test (*p* = 0.05).

Fracturability is an instrumental texture parameter that gives an idea of crunchiness [33], a sensorial attribute perceived positively by consumers in apples [34,35]. The higher the fracturability, the higher the crunchiness. The results for this parameter are shown in Figure 3. Samples treated with NTP before packaging (BL and BH) presented the lowest values, a finding similar to a previous study by [17] on fresh-cut apples treated at 15 kV, 10 min, 12.7 kHz. In that study, NTP treatment reduced the linear distance measured in the puncture test of the treated apple pieces. The authors observed a biofilm-like covering on the treated samples, possibly resulting from damage to superficial cells caused by oxidant radicals generated by NTP. This microstructural modification could explain the reduction in linear distance.

On the other hand, both control samples and those treated with NTP after packaging presented the highest values, which were thereafter maintained during the refrigerated storage. This is in agreement with [36], who observed that an in-package NTP treatment (DBD 45 kV for 50 s) caused no effect on the firmness of blueberries and that this condition was maintained during refrigerated storage. In another study, Ref. [37] observed that a DBD-treatment at 13.64 kV, 70 s, 2.7 kHz on wolfberries prevented the reduction in fruit firmness during storage. This was attributed to the inactivation of microorganisms and/or to the inhibition of respiration and the derived basal metabolism. However, the application of the treatment for 2 min started to cause a reduction in fruit firmness, likely due to tissue damage. It is important to be reminded that the decrease in firmness of the fresh fruit that commonly occurs during postharvest may be related to the water loss, the augmented pectinase activity, and the damage to the internal structure of the fruits. In the present work, although there was no effect on the fracturability of samples after the in-package treatment, apple slices treated before packaging presented a reduction in this parameter when compared to the other samples.

### 3.4. Total Phenols

Table 2 shows the total phenol concentration in apple slices subjected to the different treatments over the storage period. On day 1, no significant differences were observed among treatments; however, samples packaged in the high-barrier film tended to show higher values. On day 7, the CH and AH samples exhibited the highest concentrations, differing significantly from the BH samples, and on day 14, from the BL samples. In general, the content of total phenols was not affected by the day of storage, except for BH samples, where a significant decrease was observed. Ref. [36] reported that blueberries treated with a DBD system at 45 kV for 50 s maintained the total phenol content in fruits stored for 10–25 days while the levels of these compounds in the control groups decreased with the increased storage time. However, in our study, the preservation of the total phenols content in the samples during storage was mainly attributed to the packaging in films with a high barrier to oxygen (H), whereas the NTP treatment had no effect when applied after packaging. Ref. [38] also reported the maintenance of total phenol content during refrigerated storage in red currant following DBD treatment at 20 kV, 15 kHz, and 300 W, although in that study the treatment was applied only before packaging.

An outstanding study from [39] showed, in model solutions, that the main reactions of polyphenols exposed to NTP were oxidation, nitration, and polymerization. These reactions could lead to a reduction in total phenol content. However, the UV radiation and reactive species generated by NTP may also act as elicitors, inducing a stress-defense response in plant tissues [40]. Therefore, NTP could also cause an increase in total phenol content through the activation of phenylalanine ammonia-lyase, a key enzyme in their synthesis. In addition, the disruption of cell walls could enhance the extraction of total phenols [41].

Therefore, the absence of significant differences in total phenol content between treated and control samples suggests either that the NTP treatments had no significant impact or that a balance may exist between the synthesis of new compounds and the breakdown of existing ones. Further studies are needed to elucidate the reasons for these results by investigating the underlying metabolic responses in the fruit that NTP may trigger or inhibit. Important targets include the ascorbate–glutathione cycle, antioxidant enzymes (ascorbate peroxidase, glutathione reductase, superoxide dismutase, catalase, and relevant peroxidases), and key metabolites such as reduced and oxidized glutathione.

### 3.5. Antioxidant Capacity

Table 2 shows the antioxidant capacity of the apple slices subjected to the different treatments and different days of storage as measured by different methods (DPPH, ABTS and FRAP). For the DPPH method on day 1, the AH samples showed an antioxidant capacity higher than the other treatments, although a significant difference was only observed when compared to the samples packaged with a low oxygen barrier (approximately 60% lower capacity in the last ones). However, this difference was no longer maintained after day seven. In strawberries treated at 60 kV, 50 Hz for 10 min and packaged in low density polypropylene bags, an increase (14.5%) in antioxidant capacity (by DPPH method) was also detected just after treatment [23]. In addition, Ref. [3] reported in fresh-cut strawberries treated by a DBD-treatment at 45 kV for 1 min applied after packaging, an 18% increase in antioxidant capacity after 3 days of refrigerated storage, as measured by DPPH method.

In the present study, BL apple samples differed significantly from the control on day seven and showed lower antioxidant capacity. By day 14, these samples exhibited significantly lower antioxidant capacity than all other treatments. Ref. [30] reported a similar effect of NTP treatments in apple cubes directly exposed (without packaging) to a DBD-treatment at 20 kV, 50 Hz for 15 min. They observed a significant reduction in antioxidant capacity by DPPH method in comparison with control apple cubes. Contrarily, in our work, samples treated with NTP after packaging (AH and AL) showed a significant increase in antioxidant capacity during storage.

A trend similar to that observed with the DPPH method was found for ABTS and FRAP assays on day one, but the differences in this case were not significant. On day seven, the samples treated before packaging showed lower values than CL samples, regardless of the packaging, while on day 14 the BL samples had the lowest values. In general, no significant differences were found during storage in antioxidant capacity measured by these methods for the samples of the different treatments, except for AL samples, which showed a significant increase when measured by the ABTS method. In the case of wolfberries, Ref. [37] informed a slight increase (3%) in antioxidant capacity as measured by ABTS method after a DBD-treatment at 13.84 kV, 2.7kHz, 70 s on the second day of refrigerated storage. When treated samples were measured by DPPH, the authors determined that antioxidant capacity was not increased but was more effectively maintained than controls over a 6-day refrigerated storage period. They suggest that NTP treatment could generate an abiotic stress to the fruit tissue able to induce the accumulation of antioxidant compounds. Additionally, NTP in minimally processed fruits may also inhibit oxidase activity, preventing the degradation of antioxidant compounds. It is important to highlight that, although different results have been reported for the antioxidant capacity of fruits treated with NTP, an increase in antioxidant capacity has generally been observed when the product was treated in-package [3,14]. However, direct treatment of unpackaged fruit often resulted in either a decline in antioxidant capacity or no significant differences [42,43,44].

### 3.6. Sugars

The main soluble sugars generally found in fruit are sucrose, glucose, and fructose. These sugars, which are also the predominant carbohydrates in apple varieties such as Granny Smith, Pink Lady, and Jonagold, are directly related to fruit sweetness and are therefore regarded as essential quality indicators for minimally processed apples and apple juice [1]. In addition, sugar content in fruits influences not only sweetness but also the synthesis of other metabolites, such as acids, carotenoids, aromatic compounds, and other nutritional components [26].

Table 3 shows the concentration of sugars (sucrose, glucose, and fructose) of the apple slices subjected to different treatments and different days of storage.

On day one, the combination of NTP treatment and low oxygen barrier packaging tended to reduce sucrose content. Notably, samples treated with plasma and packaged with L differed significantly from samples CH, which exhibited the highest sucrose levels. On day seven, there was a greater effect of cold plasma when treatment was applied before packaging, with BH and BL being the samples with the lowest sucrose content. On day 14, this trend was maintained, although the significant differences among samples were no longer maintained. In all cases, the sucrose content decreased with storage, with this diminution being statistically significant in all the treatments except for the samples treated with NTP after packaging, regardless of the type of packaging. In the case of CL, although the sucrose content decreased on day seven, there was thereafter a significant increase on day 14 of refrigerated storage.

In the case of glucose concentration, the samples treated with plasma before packaging tended to have on day 1, a higher concentration than those treated after this process. By day 7, control samples packaged in the low oxygen barrier film showed the lowest concentration, while AH had the highest value. The glucose content of AH significantly differed from that of the BH and CL samples. Curiously, on day 14, the AH and CL samples had the lowest glucose values. During storage, the AH samples showed an increase in glucose concentration on day 7, and then a decrease on day 14. CH samples showed a similar trend. On the other hand, CL samples showed a decrease in glucose concentration during storage, while the rest of the treatments were rather unaffected.

On day 1, fructose content was lowest in low-oxygen-barrier samples treated with NTP, regardless of whether treatment was applied before or after packaging. However, because fructose content decreased during storage in samples packaged with the high-barrier film, especially in BH, whereas no such decrease was observed in low-barrier samples, the latter no longer differed from the controls or high-barrier samples by day 7. Nevertheless, by day 14, AL samples exhibited the lowest fructose concentration.

Sugars represent an important energy source in fruit metabolism. Fluctuations in sucrose concentration may result either from its formation through starch hydrolysis, leading to an increase, or from its conversion into the monosaccharides glucose and fructose through disaccharide hydrolysis, leading to a decrease. The concentration of simple sugars increases as sucrose is hydrolyzed, although these sugars may also be consumed in metabolic pathways such as respiration and the phenylpropanoid pathway, resulting in a decrease [45].

Ref. [1] observed in apple cubes that the concentration of fructose and glucose decreased after DBD plasma application when compared to control samples. Thus, while glucose and fructose can be generated from sucrose, these two compounds can also be consumed in other reactions. According to these authors, samples still have viable cells and viable enzymes, which may respond to reactive oxygen species (ROS) generated by the DBD plasma application, via the activation of the phenylpropanoid mechanism. Furthermore, since air plasma generates singlet oxygen and nitrogen radicals, these compounds can react with fructose and glucose, enhancing the reduction in these sugars. The same authors reported that DBD plasma application significantly reduced the sweetening power in apple juice, rendering juice with a lower total sugar content and therefore a lower sweetness. Interestingly, this product could well be commercialized as a low sugar content alternative. In the case of blueberries, the sugar content of fresh fruit typically increases initially and then decreases during the storage period. Ref. [26] found that the sugar concentrations of blueberries subjected at 6, 8 and 10 min air DBD cold atmospheric plasma treatment (36 V 1.8 A) were 51.6%, 82.2%, and 61.5% higher, respectively, compared to that of the control. While sugar content in the control reached a maximum on the fourth day of storage and then decreased, this parameter showed a slow but steady increase in the groups treated for 6–10 min, followed by a decrease that occurred later than in the control samples. These results indicate that the NTP treatment increases the sugar content and delays the onset of the sugar content decrease during storage at ambient temperature for these fruits.

Since stress responses are present in all biological systems, air cold plasma, as an environmental stressor, may induce stress responses in biological systems. Working with blueberries, Ref. [36] also found that NTP treatment (in-package DBD atmospheric air cold plasma at 45 kV for 50 s) was able to suppress the decrease in soluble sugars during 25 days of refrigerated storage.

### 3.7. Ascorbic Acid

Ascorbic acid is an important nutritional component of fruits, acting as vitamin C, and also plays a key antioxidant role. Table 3 also shows the concentration of ascorbic acid in the apple slices subjected to different treatments and days of storage. In samples subjected to NTP treatments after packaging, no significant decrease in ascorbic acid content was observed on day 1. Moreover, under the AH treatment, samples showed the highest content of this compound, differing significantly from all other treatments with the same packaging. On day 7, these samples also differed significantly from all treatments, exhibiting the highest ascorbic acid content. On day 14, no significant differences were observed among treatments. In general, no differences were found among the different samples during storage, except on day 14, when BH and AL samples increased, while AH samples decreased the content of this metabolite.

Ref. [26] observed that ascorbic acid content in blueberries treated with in-package DBD (atmospheric air, 36 V, 1.8 A) initially increased and then decreased during storage. After 16 and 20 days of storage, ascorbic acid content in the 2- to 8 min treatment groups were higher than in the control groups, reaching up to a 1.5-fold increase in the 4 min treatment. In the control group, this metabolite reached its maximum value after 8 days of storage. These results indicate that DBD plasma increased ascorbic acid content in blueberries and prolonged the preservation of the active form of this compound. The authors considered that NO was one of the excited species generated by cold plasma, which could regulate the ascorbate-glutathione cycle and increase the dehydroascorbate reductase activity. Dehydroascorbic acid is an oxidized form of ascorbic acid, which can be converted to ascorbic acid by the enzyme dehydroascorbic acid reductase [46]. Therefore, the variations in ascorbic acid content might be due to the rate of regeneration. An increase in ascorbic acid occurs when the rate of regeneration by the ascorbate-glutathione cycle surpasses the rate of its decay through reactions with other plasma-generated reactive species. Contrarily, when the rate of decay exceeds the rate of regeneration, the ascorbic acid content is reduced.

### 3.8. Light Microscopy

**Figure 4:**
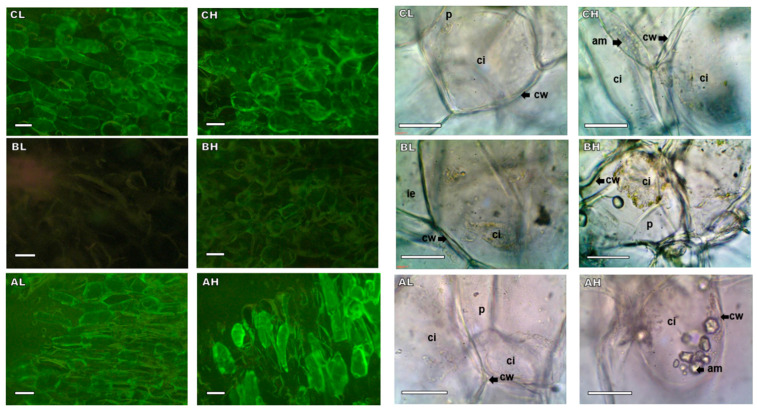
Parenchymal tissue from minimally processed apples: Cell viability (**left** side) and light microscopy (**right** side). Samples analyzed after 4 days of storage at 4 °C. References: am, amyloplasts; ci, cytoplasm; ie, intercellular space; p, space between wall and cytoplasm resulting from plasmolysis; cw, cell wall. Bars: Cell viability: 200 µm; Light microscopy: 50 µm.

Figure 4 shows the micrographs of apple tissues corresponding to the fruit subjected to the different treatments evaluated. The cellular characteristics of the sample surfaces were similar or even identical to those found in the internal part of the sample. For simple comparation, Figure 4 only includes one section per treatment. The cell walls were well preserved in all samples and showed no deformation. Generally, the parenchymal tissue maintained the shape of the cells and their intercellular spaces. However, microscopic observations of the samples treated before packaging (BL and BH) exhibited partial or complete plasmolysis of cells, with disorganization of the cytoplasm. A similar finding was reported by [47] in banana slices treated with plasma generated with ambient air at 40 V, 6 A, 10 kHz for 2 min. These authors informed damage of the cellular structure with the loss of cell content and the rupture of cell membranes in treated samples. Other side effects such as the generation of cracks and fissures were also observed and attributed to an etching effect, which occurs through physical changes and chemical reactions. Ref. [48] reported in grapes the appearance of cavities, intracellular spaces, and micro-holes in the cellular structure, mainly caused by reactive species produced by a gliding arc (50 Hz, 300 W, and 27 kV), and applied for 1 min.

In the remaining samples, parenchymal tissue cells were turgid, with organized or slightly plasmolyzed cytoplasm. Similar findings were reported in our previous study [14] on apple slices treated with in-package DBD plasma for 1 and 3 min under similar conditions. Post-packaging NTP applications show a suitable preservation of the microstructure in both packaging films, while pre-packaging applications caused partial tissue damage.

### 3.9. Viability Test

Figure 4 shows the micrographs of the viability test for the apple tissues subjected to the different treatments, as determined by the Fluorescein Diacetate (FDA) method. This method distinguishes cells with an active metabolism, as revealed by the cleavage of FDA, a nonfluorescent fluorescein analog. This compound is able to cross the cell membrane, where esterase enzymes cleave the diacetate group, producing a fluorescent product that remains in cells with intact membranes [49]. Control samples can then be compared with treated samples to visualize the effect of the treatments on the viability of the tissues. The intense fluorescence in control samples is indicative of the viability of the cells without processing. In turn, samples treated before packaging present a less intense fluorescence, with distinguished dark zones. Probably, this effect was due to the loss of membrane integrity of some cells, because of the effect of the direct cold plasma treatment. Most cells of samples treated by the other treatments (post-packaging) were viable, according to this test. It seems that the post-packaging application of NTP better preserved cell viability. To the best of our knowledge, few works have previously studied the effect of non-thermal technologies on the viability of tissues, and no study was found focusing on the effect of NTP treatments on fruit tissue viability. If the tissue fluoresces, this indicates the presence of active intracellular esterases and intact cell membranes. Intact cell membranes allow the maintenance of an osmotic difference between the inside and outside of the cells. Changes in membrane integrity, from partial to complete permeability, can lead to significant alterations in tissue architecture and texture, which are key factors in consumer acceptance of food [50].

In minimally processed peaches subjected to high hydrostatic pressure (HHP) processing (600 MPa-5 min), Ref. [21] observed a total absence of fluorescence in treated tissues, which evidences the total loss of cell viability because of the HHP treatment. In another study, Ref. [51] used the FDA method to evaluate the effect of pulsed electric fields on strawberries and kiwifruit. Although the application of low electric field strength (100 V/cm) adequately preserved the tissue viability, a high intensity treatment (200 V/cm) caused tissue damage and loss of viability, probably due to electroporation. In turn, in a study evaluating the effect of different processing operations on cranberries, Ref. [52] reported that samples prepared from whole and cut cranberries retained viable cells throughout the tissue, whereas blanching completely impaired cell viability. Similarly, samples subjected to blanching followed by ultrasound (US) treatment (21 kHz for 30 or 60 min) also showed cell death.

As the viability test uses tissue fluorescence to readily assess metabolic activity after novel treatments, it is expected that more studies evaluating fruit tissue viability after the application of novel technologies such as cold plasma will become available in the future.

## 4. Conclusions

In this work, two different ways of applying cold atmospheric plasma were evaluated: Direct application and in-package application. In addition, two different packaging films were tested (one with high barrier to oxygen and one with low barrier to oxygen) to define which of the NTP applications and packaging better preserved apple slices during refrigerated storage. Regarding chromatic and textural parameters, the only samples that showed development of browning and lower crunchiness were the ones treated before packaging. Samples treated with NTP after packaging preserved better the microstructure and tissue viability of the slices. After analyzing different microbiological, physicochemical, biochemical, nutritional, and structural parameters, we found that the combination of NTP applied after packaging with the high-barrier film (AH) was the most suitable for maintaining fruit quality.

## Figures and Tables

**Table 1:** Treatments for minimally processed apples.

Packaging Film	Non Thermal Plasma Treatment (DBD Ambient Air 35 kV, 50 Hz, 1 min)		
	Without Treatment (Control)	Before Packaging (B)	After Packaging (A)
High oxygen barrier (H)	CH	BH	AH
Low oxygen barrier (L)	CL	BL	AL

**Table 2:** Total phenol content and antioxidant capacity by the ABTS, DPPH, FRAP methods of minimally processed apples treated at different non-thermal plasma (NTP) treatments during storage at 4 °C.

	Days of Storage at 4 °C		
	1	7	14
Total phenols (g eq. Gallic Acid/kg fresh fruit)			
CH	0.57 ± 0.09 aA	0.62 ± 0.01 aA	0.60 ± 0.06 aA
AH	0.73 ± 0.07 aA	0.63 ± 0.02 aA	0.60 ± 0.03 aA
BH	0.72 ± 0.07 aA	0.44 ± 0.04 bB	0.55 ± 0.05 aAB
CL	0.52 ± 0.01 aA	0.56 ± 0.06 abA	0.51 ± 0.04 abA
AL	0.50 ± 0.05 aA	0.50 ± 0.04 abA	0.54 ± 0.01 aA
BL	0.51 ± 0.07 aA	0.51 ± 0.08 abA	0.39 ± 0.05 bA
Antioxidant capacity DPPH method (mMol eq. Trolox/kg fruit)			
CH	2.3 ± 0.4 abA	2.6 ± 0.1 abA	3.5 ± 0.3 aA
AH	3.4 ± 0.6 aAB	2.5 ± 0.1 abB	3.6 ± 0.2 aA
BH	3.1 ± 0.1 abA	2.9 ± 0.1 abA	3.3 ± 0.5 aA
CL	2.2 ± 0.1 bA	3.2 ± 0.6 aA	3.0 ± 0.3 aA
AL	2.0 ± 0.3 bB	2.9 ± 0.3 abAB	3.3 ± 0.2 aA
BL	2.1 ± 0.3 bA	2.1 ± 0.2 bA	2.0 ± 0.3 bA
Antioxidant capacity ABTS method (mMol eq. Trolox/kg fruit)			
CH	2.3 ± 0.4 aA	2.8 ± 0.1 abA	3.3 ± 0.1 aA
AH	3.2 ± 0.3 aA	2.7 ± 0.1 abA	3.2 ± 0.1 abA
BH	3.1 ± 0.2 aA	2.4 ± 0.2 bA	3.0 ± 0.3 abA
CL	2.4 ± 0.2 aA	3.1 ± 0.4 aA	2.5 ± 0.1 bcA
AL	2.3 ± 0.2 aB	2.7 ± 0.2 abAB	2.9 ± 0.1 abA
BL	2.4 ± 0.4 aA	2.2 ± 0.2 bA	2.0 ± 0.3 cA
Antioxidant capacity FRAP method (mMol eq. Trolox/kg fruit)			
CH	1.8 ± 0.3 aA	2.1 ± 0.1 abA	2.3 ± 0.1 aA
AH	2.6 ± 0.5 aA	2.0 ± 0.1 abA	2.3 ± 0.1 aA
BH	2.3 ± 0.1 aA	1.7 ± 0.2 bA	2.2 ± 0.3 aA
CL	1.7 ± 0.1 aA	2.3 ± 0.4 aA	1.9 ± 0.1 abA
AL	1.7 ± 0.2 aA	2.0 ± 0.1 abA	2.1 ± 0.1 abA
BL	1.8 ± 0.3 aA	1.6 ± 0.2 bA	1.4 ± 0.3 bA

Data are expressed as means + standard error (*n* = 3). For each storage time, the means at different treatment followed by the same lowercase letter were not significantly different according to Duncan’s test (*p* = 0.05). For each plasma treatment, means at different storage time followed by the same uppercase letter were not significantly different according to Duncan’s test (*p* = 0.05).

**Table 3:** Sugar (Glucose, fructose, and sucrose) and ascorbic acid content of minimally processed apples treated at different non-thermal plasma (NTP) treatments during storage at 4 °C.

	Days of Storage at 4 °C		
	1	7	14
Sucrose (g/kg fresh fruit)			
CH	43 ± 2 aA	31 ± 5 aAB	25 ± 2 aB
AH	32 ± 2 abA	25 ± 2 abA	28 ± 7 aA
BH	35 ± 1 abA	12 ± 2 cB	16 ± 1 aB
CL	36 ± 1 abA	22 ± 1 abcB	34 ± 1 aA
AL	28 ± 6 bA	21 ± 4 abcA	30 ± 2 aA
BL	27 ± 2 bA	14 ± 3 bcB	16 ± 5 aB
Glucose (g/kg fresh fruit)			
CH	21 ± 1 abAB	24 ± 1 abA	17 ± 2 abB
AH	18 ± 1 abB	27 ± 4 aA	13 ± 1 bB
BH	22 ± 1 aA	17 ± 1 bcA	16 ± 4 abA
CL	19 ± 1 abA	14 ± 1 cAB	13 ± 1 bB
AL	17 ± 2 bA	19 ± 1 abcA	17 ± 1 abA
BL	21 ± 3 abA	22 ± 3 abcA	22 ± 1 aA
Fructose (g/kg fresh fruit)			
CH	63 ± 2 aA	59 ± 2 abAB	53 ± 1 abB
AH	65 ± 1 aA	63 ± 3 aA	52 ± 2 abB
BH	62 ± 1 aA	47 ± 9 cC	52 ± 1 abB
CL	61 ± 1 aA	53 ± 1 bcA	59 ± 8 abA
AL	55 ± 1 bA	57 ± 2 abA	48 ± 2 bB
BL	53 ± 5 bA	59 ± 2 abA	62 ± 4 aA
Ascorbic Acid (mg/100 g fresh fruit)			
CH	3.6 ± 0.5 bcA	4.2 ± 0.1 bA	3.6 ± 0.2 aA
AH	4.5 ± 0.2 aA	4.4 ± 0.1 aA	3.9 ± 0.1 aB
BH	3.1 ± 0.1 cB	4.2 ± 0.1 bA	4.2 ± 0.2 aA
CL	4.1 ± 0.3 abA	3.8 ± 0.1 cA	3.9 ± 0.1 aA
AL	4.1 ± 0.1 abAB	3.8 ± 0.1 cB	4.1 ± 0.1 aA
BL	3.6 ± 0.2 bcA	4.0 ± 0.1 bcA	4.1 ± 0.2 aA

Data are expressed as means + standard error (*n* = 3). For each storage time, the means at different treatment followed by the same lowercase letter were not significantly different according to Duncan’s test (*p* = 0.05). For each plasma treatment, means at different storage time followed by the same uppercase letter were not significantly different according to Duncan’s test (*p* = 0.05).

## Data Availability

The original contributions presented in the study are included in the article/Supplementary Materials. Further inquiries can be directed to the corresponding author.

## Supplementary Materials

The following supporting information can be downloaded at: https://www.mdpi.com/article/10.3390/foods15132288/s1, Table S1: Browning Index (BI) of minimally processed apples treated at different non-thermal plasma (NTP) treatments during storage at 4 °C.

## Abbreviations

The following abbreviations are used in this manuscript:

NTP	Non thermal plasma technology
ROS	reactive oxygen species
DBD	dielectric barrier discharge
GAE	gallic acid equivalents
FRAP	Ferric reducing/antioxidant power
TEAC	Trolox (6-hydroxy-2,5,7,8-tetramethylchroman-2-carboxylic acid) equivalents antioxidant capacity
RAM	total aerobic mesophilic counts
SSC	Soluble solid content
CFU	colony forming units
FDA	Fluorescein Diacetate
HHP	high hydrostatic pressure
