# Supplementary material for: Cold Atmospheric Plasma: Pre- and Post-Packaging Application for Fresh-Cut Apple Preservation
Source: Foods. 2026 Jun 26;15(13):2288. doi: 10.3390/foods15132288 (PMC13362370; doi:10.3390/foods15132288)
Supplement: Supplementary file 1 [file foods-15-02288-s001.zip › foods-4202376-supplementary.pdf]

Table S1. Browning Index (BI) of minimally processed apples treated at different non-thermal plasma (NTP) treatments during storage at 4°C.

Treatment	Days of refrigerated storage		
	1	7	14
CH	17 ± 1 aB	21 ± 3 bAB	27 ± 2 aA
AH	18 ± 3 aA	18 ± 2 bA	28 ± 7 aA
BH	18 ± 2 aB	26 ± 4 abAB	29 ± 3 aA
CL	18 ± 2 aB	25 ± 1 abA	23 ± 1 aA
AL	18 ± 1 aB	23 ± 3 bAB	26 ± 2 aA
BL	23 ± 3 aA	32 ± 4 aA	31 ± 4 aA

Data are expressed as means ± standard error (n=3). For each storage time, the means at different treatment followed by the same lowercase letter were not significantly different according to Duncan´s test (p=0.05). For each plasma treatment, means at different storage time followed by the same uppercase letter were not significantly different according to Duncan´s test (p=0.05).
